# Treating frailty-a practical guide

**DOI:** 10.1186/1741-7015-9-83

**Published:** 2011-07-06

**Authors:** Nicola Fairhall, Colleen Langron, Catherine Sherrington, Stephen R Lord, Susan E Kurrle, Keri Lockwood, Noeline Monaghan, Christina Aggar, Liz Gill, Ian D Cameron

**Affiliations:** 1 Rehabilitation Studies Unit, Faculty of Medicine, The University of Sydney, Ryde, Sydney 2112, Australia; 2 The George Institute for Global Health, The University of Sydney, Sydney 2000, Australia; 3 Rehabilitation and Aged Care Services, Hornsby Ku-ring-gai Hospital, Hornsby, Sydney 2077, Australia; 4 Neuroscience Research Australia, University of New South Wales, Randwick, Sydney 2031, Australia; 5 Faculty of Nursing and Midwifery, The University of Sydney, Sydney 2006, Australia

## Abstract

Frailty is a common syndrome that is associated with vulnerability to poor health outcomes. Frail older people have increased risk of morbidity, institutionalization and death, resulting in burden to individuals, their families, health care services and society. Assessment and treatment of the frail individual provide many challenges to clinicians working with older people. Despite frailty being increasingly recognized in the literature, there is a paucity of direct evidence to guide interventions to reduce frailty. In this paper we review methods for identification of frailty in the clinical setting, propose a model for assessment of the frail older person and summarize the current best evidence for treating the frail older person. We provide an evidence-based framework that can be used to guide the diagnosis, assessment and treatment of frail older people.

## Background

Identification and treatment of frailty is a challenge for clinicians. Frailty is a common geriatric syndrome, characterized by decreased reserve and increased vulnerability to adverse outcomes, including falls, hospitalization, institutionalization and death [1,2]. The prevalence and the consequences of frailty present a considerable burden to older people, their carers, health care services and the community. Interventions designed to reduce frailty therefore have the potential for profound and widespread benefits.

Management of the frail older individual is challenging on multiple levels. Understanding of frailty has increased dramatically over the past decade, thanks to research into the biological basis of frailty and methods to define and predict the syndrome. There is no firm consensus, however, on how to assess and diagnose frailty in the clinical setting [3]. Care of frail individuals is also difficult, due to complex comorbidities, vulnerability to deterioration and increased social needs [1,4], compounded by the need for consistent ongoing management despite frequently fragmented health service delivery. A practical, evidence-based guide for clinicians is therefore needed.

There is a paucity of direct evidence guiding interventions to decrease frailty. Research has concentrated on the effect of interventions on functional and nutritional outcomes in frail older people [5-9] and application of geriatric models of care to frail older people in a variety of settings [4,10]. However, clinical improvement from the frail state is possible [11,12] and there is an urgent need for effective interventions to mitigate frailty. As in other fields, clinicians should aim to integrate the highest levels of evidence with clinical experience and patient values as suggested by Sackett and others [13,14]. A literature search identified no clinical trials that have investigated whether intervention can alter or reverse frailty. In the absence of such evidence, clinicians can be guided by clinical trial evidence answering related questions and we also draw on our collective experience in aged care and rehabilitation.

This paper aims to provide a synthesis of the current available evidence concerning interventions to decrease frailty and provide practical information on identification of frailty in clinical practice and provision of interventions to reduce frailty in the clinical setting.

## Discussion

### Identification and assessment of frailty

Screening and assessment of frailty are increasingly recommended components of the assessment of older people [10], yet there is no consensus definition of frailty. Multiple operational definitions of frailty have been proposed [15-19], and the components of the main phenotypes of frailty are well critiqued in a recent review [3]. The current literature has two primary definitions of frailty. The Frailty Phenotype [1] diagnoses people as frail if they meet pre-determined values for three or more of five criteria: weak grip strength, slow walking speed, exhaustion, weight loss, low energy expenditure. Frailty indices have been developed to quantify accumulation of identified deficits present across multiple domains (for example cognition, mood, social resources, chronic diseases), an example of which is the Frailty Index [19]. Alternate measures include combinations of instruments each measuring a single aspect of frailty [2]. There will likely be future developments in this area, and consensus on how to diagnose frailty will assist clinicians and researchers alike.

In the busy clinical setting, a single item assessment tool may indicate whether more comprehensive assessment is required in order to make a diagnosis of frailty. Reduced gait speed is clearly predictive of future vulnerability [20,21] and although cut-off speeds are contentious, 0.8 m/sec was proposed as the result of a recent systematic review [21] and seems a useful starting point for further validation studies. In the event that potential for frailty is identified, we suggest referral for multi-disciplinary assessment where resources are available. The simple assessment form provided in Additional file 1 (Frailty assessment form) is based upon comprehensive geriatric assessment and captures frailty using the Frailty Phenotype [1]. Completed during routine comprehensive geriatric assessment, this form may also be used to count deficits in health, to formulate a frailty index using the standardized procedure of Searle and colleagues [22], as has been done with previous assessment tools [23,24].

**Figure 1:**
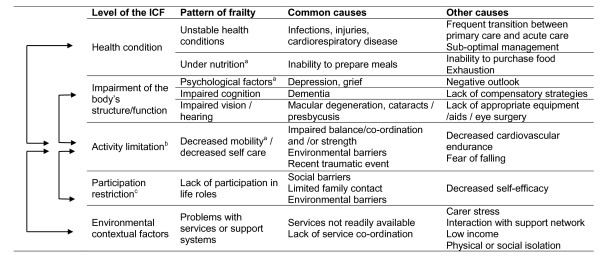
**Important factors to assess in the frail patient and the interactions between them**. ^a^Captured in the Frailty Phenotype; ^b^Defined as difficulty experienced by an individual when executing activities (International Classification of Functioning (ICF)[26]); ^c^Defined as problems experienced by an individual in their involvement in life situations (ICF).

Once frailty is identified, management is guided by multidisciplinary assessment using the principles of Geriatric Evaluation and Management (GEM) [10,25], while specifically targeting the problems associated with frailty. In order to overcome the problems associated with inconsistent definitions, we suggest frailty can manifest in a number of recognizable patterns that may be useful for guiding evaluations and intervention. Important factors routinely assessed and common interactions between them, are described in Figure 1, using the framework of the International Classification of Functioning, Disability and Health (ICF) [26].

Suggested principles of assessment in older people diagnosed as frail are: (1) Comprehensive, interdisciplinary assessment of physical, emotional, psychological and social factors, and support mechanisms [25]. This may be time consuming due to comorbidities, polypharmacy, pain and disability resulting from impaired vision, hearing, speech and cognition. (2) Assessment of psychological and social factors that are potential barriers to implementation, uptake and adherence with the intervention. (3) Regular reassessment, particularly following an illness or injury, to detect changes in needs and ensure timely modifications of care provision.

### Principles of intervention to reduce frailty

The lack of consensus regarding the definition and components of frailty will influence clinicians' approach to intervention. For example, the Frailty Phenotype [1] identifies five potential deficits that may be targeted with treatment while an extensive frailty index will yield a more comprehensive picture of the individual, with the potential for broader intervention [23]. We propose clinicians should specifically identify and target the dimensions of frailty identified in the Frailty Phenotype, as highlighted in Additional file 2 (Additional recommendations for treatment of the frail older person), but also incorporate GEM to ensure the complex picture present in the frail older person is comprehensively addressed, as discussed by Rockwood and Mitnitski [23]. GEM decreases functional decline and the use of home health care services in older people susceptible to recurrent hospitalization [10]. Intervention delivered to the frail older person should accord with the best available evidence for each problem identified at assessment. The challenge is to implement the evidence in the real-life setting of a health system, and to co-ordinate management of interventions addressing multiple problems, in a population vulnerable to adverse outcomes.

We propose that intervention be based upon six premises: (1) Frailty can be mitigated. (2) Support will need to be individually tailored to address each person's underlying problems as well as the contextual factors that influence their condition and response to intervention. (3) Support has to be delivered over a long period and systems must facilitate consistent management in the presence of acute health events. (4) Intervention aims to improve physical, cognitive and social functioning and extend frail older individuals' length of time in independence and self-management, living in their preferred setting; in addition, intervention aims to decrease vulnerability to adverse outcomes, in particular falls, injury, hospitalization and institutionalization. (5) Most frail older people should be encouraged and supported to adhere to their intervention plan. (6) It is important to recognize the needs of family and/or carers and to engage with them.

### Implementation of interventions to reduce frailty

#### The content

Interventions that address the common patterns of frailty are outlined in Table 1. Each intervention is drawn from the highest quality evidence available for an assessed problem and is consistent with management deliverable in clinical practice. Medical management of unstable health conditions may include the use of clinical guidelines, review of medications, intervention to increase compliance, and referral to other clinicians. Nutritional state, mood, cognition, vision and hearing should be addressed according to guidelines developed for older people, with interventions taking into account the susceptibilities associated with frailty, for example the side effects of antidepressant medication [27]. Decreased mobility and falls risk can be effectively addressed in the frail older person via appropriately designed exercise and home safety interventions [28,29], and targeting the causes of decreased participation in life roles can improve involvement at the societal level [30,31]. The frailty management plan should be coordinated over a specified period, with case co-ordination and early and ongoing engagement of carers [25], to minimize problems with services and support systems. Most importantly, treatment needs to be personalized and adapted to the goals, capacity and context of the individual.

**Table 1:** Interventions and guidelines to address the common patterns of frailty in the clinical setting

Pattern	Screening and assessment	**Intervention (See Additional file **2** for more detailed recommendations)**
Unstable health conditions	Medical assessment	Medical management may include geriatrician review, medication review, intervention to increase compliance, referral for follow-up of medical conditions, for example memory clinic, continence clinic.
		Specific information for common health conditions is provided in Additional file 2.
Under nutrition	Setting appropriate screening, e.g. Mini Nutritional Assessment [48]	Referral to a dietician for nutritional support, which may include: education about foods rich in energy and protein, nutrition advice about general healthy eating and benefits of regular exercise to improve health and overall wellbeing, and nutrition support. The NICE clinical guideline 'Nutrition Support in Adults' provides high quality evidence for oral nutrition support in adults with malnutrition http://guidance.nice.org.uk/CG32 (Chapter 8 in particular).
Psychological factors	Geriatric Depression Scale (short form) [49]	The Victorian Government Health Information toolkit for depression http://www.health.vic.gov.au/older/toolkit/06Cognition/03Depression/index.htm Frail older depressed patients are particularly susceptible to side effects of antidepressant medication [27]. Antidepressant medication should therefore not be used as front line therapy. Antidepressant medication is effective in the treatment of older people [50], and a comparison of treatments is outlined in the Cochrane review by Mottram and colleagues [27]. The NICE clinical guideline 'Occupational therapy interventions and physical activity interventions to promote the mental wellbeing of older people in primary care and residential care', http://guidance.nice.org.uk/PH16
Impaired cognition	Mini Mental Status Examination (MMSE) [51] and/or informant questionnaire (IQ Code) [52]	The NICE clinical guideline, 'Dementia: Supporting people with dementia and their carers in health and social care', http://guidance.nice.org.uk/CG42
Impaired vision/hearing	Brief clinical assessment	Referral for specialist medical assessment Facilitate liaison with local/national foundation for blindness and low vision, for aids and advice Facilitate self-management of aids for vision/hearing
Decreased mobility	Timed 4 m walk Timed Up and Go Lower limb strength: Timed sit to stand Balance: 4-point balance test Falls risk: Physiological Profile Assessment [53]	Appropriately designed exercise interventions are effective in preventing falls in older people living in both the community [28] and nursing care settings [29]. Exercise should be ongoing, challenge balance and be undertaken at least two hours per week [43]. Home safety interventions reduce falls rate in this high-risk frail group, and multifactorial falls assessment and intervention are also effective [28]. The team should intervene or refer to appropriate disciplines. Strategies to facilitate behavior change to enhance participation in intervention programs are outlined in the NICE Guidance 'The most appropriate means of generic and specific interventions to support attitude and behaviour change at population and community levels', http://www.nice.org.uk/PH6[33]. We also encourage the implementation of the Recommendations on physical activity for health for older Australians http://www.health.gov.au/internet/main/publishing.nsf/Content/phd-physical-rec-older-guidelines[54].
Lack of participation in life roles	Clinical assessment	Barriers to participation should be assessed. Randomized controlled trials have demonstrated increased participation with intervention targeting risk factors, such as modification of the home environment [30] and specific training of community interactions [31]. Setting individualised goals and tailoring interventions to meet these goals may also be effective. Enlist help of significant others/carers to encourage goal attainment.
Problems with services or support systems	Clinical assessment	There should be early and ongoing engagement with support and education of formal and informal carers [25]. Caregivers and family should be taught about frailty, interventions to optimize function, and be involved in planning and development of management plans. Provision and co-ordination of services, with preference given to packages of care, followed by single services, followed by a residential aged care facility. The case co-ordinator must ensure the frail individual and their family/carers understand the services provided and how to promptly access greater assistance in times of increased need. Advice for assisting carers is provided in the NICE clinical guideline 'Dementia: Supporting people with dementia and their carers in health and social care' http://guidance.nice.org.uk/CG42, Section 1.11.

#### Focus on increasing adherence

Patient adherence has been shown to increase the effectiveness of health interventions [32]. Application of the principles of behavior change [33] to frail individuals involves acknowledging that components of frailty can be treated and specifying the link between the intervention and outcome. The targets of the intervention should be described to the individual, the choice of intervention justified, and the method of evaluation discussed, as agreement between the patient and primary care physician regarding health care significantly increases adherence to geriatric treatment plans [34]. Individuals should be supported, motivated, empowered, and given assistance to develop both goals and the strategies to achieve them. Potential barriers to adherence should be assessed and addressed, with a particular reference to engagement of social support systems.

#### The team

The interdisciplinary team must be trained and experienced in the care of older people. The team working with the frail older person includes multiple health professionals with close links to the general practitioner who provides primary healthcare, and other service providers. The nature of frailty necessitates the availability of a physiotherapist to address mobility related function, including strength, balance and falls-prevention and a dietician for nutritional assessment and management. In our experience, a geriatrician/rehabilitation physician is generally necessary. In the Australian setting personnel require close links with the local Aged Care Assessment Team to facilitate care package review, and an awareness of local services such as day centers, community groups, carer supports, providers of meals, transport and in-home assistance, podiatry and services for the hearing and vision impaired. The team should have access to other allied health staff as required, and given the significant role that family/carers play in supporting frail older persons in the community [35,36], it is recommended the team have access to a health professional with carer experience when appropriate. The optimal structure and practice of effective teams are not well established and results of team-based trials must be generalized to the clinical setting with caution. Known characteristics of effective teams should be incorporated in the team structure, for example there is consensus that inter-professional collaboration should be promoted [37]. Strategies include employing policies and systems that: facilitate a shared decision-making process, encourage communication regarding patient care and reduce duplication of patient information and testing.

#### Advocacy and coordination

Literature suggests multifaceted interventions including case co-ordination improve function in vulnerable older people [38,39], however the effect of case management alone has focused on health care utilization and is less conclusive [40,41]. It is not uncommon for the frail older person to move between settings, and receive multiple services from varied service providers over the course of a single year. Case coordinators provide long-term advocacy and coordination of healthcare services to these high-risk individuals. A coordinator anticipates needs and reduces the number of interfaces encountered by the patient and his/her family, thus reducing the potential for confusion due to poor communication. Collaboration with the patient, general practitioner, carers, family and service providers ensures the older person and family are always aware of their care plan and options. A recent review of case management for long-term conditions found case management is more effective if the interventions and procedures are well-defined, caseload size is appropriate (for example there is adequate time for patient monitoring and review), and case-management practice allows for continuity of care in terms of assessment, monitoring, review and management of resources [42]. In addition, targeting those patients with identified problems that require intervention may increase the effectiveness of the intervention [39].

#### Duration and frequency

The fluctuating care needs of frail older people require the provision of long-term management with flexible delivery. We propose that frailty intervention be provided until end of life, or a time when specialist nursing or palliative services are indicated, or when the person moves to a residential aged care facility permanently. Capacity to modify the frequency of appointments is essential, due to vulnerability to deterioration. For example, to improve balance, regular appointments are necessary to ensure at least two hours of exercise occur per week on an on-going basis [43]. More frequent intervention may be required to optimize outcomes following deteriorations.

#### Resources

Regular case conferences facilitate management by the team and assist in coordinating strategic involvement of external health professionals and service providers. The case coordinator requires long-term, centralized access to information about admission and service provision. It is also necessary to have access to prompt specialist medical care via the primary care provider, such as medication review and continence and memory clinics.

#### Structure of health service

The challenge of delivering an effective, co-ordinated intervention to decrease frailty, over a prolonged period of time and in times of crisis, is substantial. The challenge is to embed an assessment and intervention program within the existing health care system which is often fragmented [38]. We propose that the optimal system would be an augmentation of an existing aged care health service, where patients are routinely assessed for frailty by primary or secondary health services, and a tailored program is delivered to decrease frailty in older people who are frail and are likely to be adherent to treatment. It may be practical for frail people to be identified in general practice based upon hospital discharge reports, health assessments for people more than 75 years old, or reports of difficulty coping at home.

#### Additional recommendations for care of frail older people

Detailed recommendations concerning evidence-based intervention are presented using the ICF categories most relevant to frail older people [see Additional file 2: Additional recommendations for treatment of the frail older person]. This approach may not be realistic in resource-poor settings, however once more is understood about frailty and its modification, the most cost-effective interventions can be determined. There is a need for randomized controlled trials to evaluate the effect of intervention on frailty, in order to determine whether frailty can be reduced or reversed. To our knowledge, at least three trials measuring the outcome of frailty are in progress [44-46].

In the future it may also be useful to develop a formal ICF core set for frailty to identify subsets of the ICF categories most applicable to frail people, according to the perspective of the individual and based upon evidence and international consensus [47]. Already developed for chronic conditions such as osteoarthritis and chronic pain, we propose an ICF core set describing the spectrum of problems characteristic of frail older people, which would aid assessment and documentation of health and functioning in research and the clinical setting.

## Summary

It is possible to identify frail older people in the clinical setting and to deliver an intervention program targeting the components of frailty, in accordance with the best available evidence for each problem identified at assessment. We have outlined the challenge of implementing the evidence in the setting of a public health system, and have described how it may be possible to co-ordinate management of interventions addressing multiple problems, in a population vulnerable to adverse outcomes.

## Abbreviations

GEM: Geriatric Evaluation and Management; IQ Code: Informant questionnaire; ICF: International Classification of Functioning, Disability and Health; MMSE: Mini Mental Status Examination.

## Competing interests

The authors declare that they have no competing interests.

## Authors' contributions

NF participated in design and coordination of the project, contributed to the content of the manuscript and drafted the manuscript. CL, CS and SRL contributed to the design and content of the manuscript. KL, NM, LG and CA contributed to the content of the manuscript. IC and SK developed the concept of the study and contributed to its design and content. All authors contributed to revisions and approved the final manuscript.

## Authors' information

NF is a Physiotherapist and a PhD candidate investigating participation in life roles by frail older people.

CL is a Physiotherapist working in aged care and rehabilitation and delivered intervention on the Frailty Intervention Trial.

CS is a Senior Research Fellow at the George Institute for Global Health, The University of Sydney. She is an Honorary Senior Research Associate at Neuroscience Research Australia, The University of New South Wales.

SL is a Senior Principal Research Fellow at Neuroscience Research Australia, The University of New South Wales, Sydney, Australia.

SK is a Geriatrician and Clinical Director of Rehabilitation and Aged Care Services at Hornsby Ku-ring-gai Hospital.

KL is a Registered Nurse and Research Consultant, co-ordinating recruitment and data management on the Frailty Intervention Trial.

CA is a Registered Nurse and PhD candidate studying carers of frail older people.

NM is a Research Fellow at The University of Sydney and Research Program Manager for a NH&MRC Program Grant 'Transition Care: Innovation and Evidence'.

LG is in the final stage of a PhD. She has worked as a health professional at the direct service provision, the health system design, the policy and consulting levels.

IC is a Consultant Physician in Rehabilitation Medicine. He is the first named investigator for the NH&MRC Program Grant 'Transition Care: Innovation and Evidence' of which a randomized controlled trial of frail older people is one of a number of studies.

## Pre-publication history

The pre-publication history for this paper can be accessed here:

http://www.biomedcentral.com/1741-7015/9/83/prepub

## Supplementary Material

Additional file 1 **Frailty assessment form**. Single page template to guide assessment of the frail older person. Click here for file

Additional file 2 **Additional recommendations for treatment of the frail older person, classified using the ICF framework**. Table illustrating an extensive list of problems seen in the frail older person and evidence based intervention to address each of these problems. Click here for file
