# Supplementary material for: Treating frailty-a practical guide
Source: BMC Med. 2011 Jul 6;9:83. doi: 10.1186/1741-7015-9-83 (PMC3146844; doi:10.1186/1741-7015-9-83)
Supplement: Additional file 1 — Frailty assessment form. Single page template to guide assessment of the frail older person. [file 1741-7015-9-83-S1.PDF]

Additional file 1 - Frailty assessment form.

Name		Date of birth	MRN
Address			Phone
Next of kin	Phone	General practitioner	Phone

Residence/access	Source of income
Lives alone/family<br><br>Social support & relationships	Community support<br><br>Satisfaction with services

Primary carer	Hip protectors	Personal alarm
Health conditions	Medications<br><br><br>Medication compliance	

History of falls	Fear of falls? Yes/No

Body structure & function	Activities & Participation	Equipment
Oriented to (circle) time/place/person<br>Cognition. MMSE score: /30	Bathing/showering	
Mood. GDS(short form): /15	Dressing	
Vision	Meals	
Hearing	Cleaning	
Pain	Laundry	
Continence	Shopping	
Range of motion	Transportation	
Strength<br><br>Grip strength ______kg<br>Grip strength<20th centile? (✓/✗)*	Amount of physical activity<br><br>*Past 3mths: no physical activity & ≥4 hrs/day sitting & ≤1 short walk/mth*(✓/✗)*	
Weight: ______kg. Unintentional weight loss ≥4.5kg in past year? (✓/✗)*	Recreation & leisure	
Height:_______m. BMI: _____ kg/m^2	Community life (e.g. clubs, church, work)	
Nutritional Assessment	Managing finances<br>Power of Attorney	

Exhaustion: "How often did you feel: Everything you did was an effort in the last week?; You could not get going in the last week?"*(Never/minimal/moderate/most of the time).*(mod/most=✓)*
Observations: posture limbs feet
Gait pattern (describe)<br><br>Timed 4m walk: _____ sec Aid used____________________ Gait speed<20th centile? (✓/✗)*
Timed up and Go: _____sec Aid used____________________ Chair height_______cm
4 Test Balance Test (seconds): Shoes on/off<br>Feet together___s Semi-tandem stand ____s Tandem stand ____s One leg stand____s
*Frailty score (Add affirmative answers to * criteria): 0=Robust, 1,2=Prefrail, 3,4,5=Frail
Primary problems
Plan
Personal factors to be considered (e.g. motivation, compliance, support)

Signed	Date	© Cameron et al 2011. Use without alteration is permitted.
