# Supplementary material for: Treating frailty-a practical guide
Source: BMC Med. 2011 Jul 6;9:83. doi: 10.1186/1741-7015-9-83 (PMC3146844; doi:10.1186/1741-7015-9-83)
Supplement: Additional file 2 — Additional recommendations for treatment of the frail older person, classified using the ICF framework. Table illustrating an extensive list of problems seen in the frail older person and evidence based intervention to address each of these problems. [file 1741-7015-9-83-S2.DOC]

***Additional file 2 - Additional recommendations for treatment of the frail older person, classified using the ICF framework.***

| **Level of ICF** | **ICF code** | **Title of the ICF domains *^a^*** | **Recommendation** |
| --- | --- | --- | --- |
| Health conditions |  | Osteoarthritis | - The NICE clinical guideline ‘Osteoarthritis’, <http://guidance.nice.org.uk/CG59> |
|  | Chronic Obstructive Pulmonary Disease | - The NICE clinical guideline ‘Chronic Obstructive Pulmonary Disease (update)’ <http://guidance.nice.org.uk/CG101> |
|  | Under nutrition *^b^* | - Referral to a dietician for nutritional support, which may include education about foods rich in energy and protein, nutrition advice about general healthy eating & benefits of regular exercise to improve health & overall wellbeing, and nutrition support. - The NICE clinical guideline ‘Nutrition Support in Adults’ provides high quality evidence for oral nutrition support in adults with malnutrition <http://guidance.nice.org.uk/CG32> (Chapter 8 in particular). |
|  | Cardiovascular disease | - The NICE clinical guideline ‘Chronic Heart Failure’   <http://guidance.nice.org.uk/CG108> |
|  | Diabetes | - The NICE clinical guidelines ‘Type 2 Diabetes’ <http://guidance.nice.org.uk/CG66> and ‘Type 1 Diabetes’ <http://guidance.nice.org.uk/CG15> |
|  | Vulnerability to adverse health outcomes | - Involvement in specific health activity, including:   - Vaccinations – influenza and pneumococcal (also see <http://www.cdc.gov/vaccines/recs/schedules/downloads/adult/2010/adult-schedule.pdf>)   - Skin checks   - Vision assessment, yearly   - Hearing assessment, four yearly   - Consider assessment of Vitamin D levels   - Medication review, including application of the Beers criteria for identifying potentially inappropriate medication use . |
| Body structures | s7700 | Bone structure  *(e.g. osteoporosis)* | - Treatment for osteoporosis is outlined in a frail population: <http://www.mja.com.au/public/issues/193_03_020810/duq11464_fm.html> |
| s730-s760 | Structure of upper extremity, trunk, lower extremity  *(e.g. kyphosis, genu valgus, hallux valgus, amputation)* | - Referral to podiatrist or orthopaedic specialist. - Orthotics may be indicated to provide support. |
| s7702 | Muscle *(e.g. sarcopenia)^b^* | - Resistance exercise can improve muscle mass and strength in older adults .  - See nutritional guidelines under ‘Under nutrition’. |
| Body functions | b1 | Cognitive functions  *(e.g. Impaired memory, problem solving)* | - Assess for and treat dementia. - Teach patient and carers compensatory strategies. - The NICE clinical guideline ‘Dementia: Supporting people with dementia and their carers in health and social care’, <http://guidance.nice.org.uk/CG42> |
| b152 | Emotional functions | - The Victorian Government Health Information toolkit for depression   <http://www.health.vic.gov.au/older/toolkit/06Cognition/03Depression/index.htm>   - Frail older depressed patients are particularly susceptible to side effects of antidepressant medication . Antidepressant medication should therefore not be used as front line therapy. Antidepressant medication is effective in the treatment of older people , and a comparison of treatments is outlined in the Cochrane review by Mottram and colleagues . - The NICE clinical guideline ‘Occupational therapy interventions and physical activity interventions to promote the mental wellbeing of older people in primary care and residential care’, <http://guidance.nice.org.uk/PH16> |
| b210-b229 | Vision functions | - Referral for specialist medical assessment. - Facilitate liaison with local/national foundation for blindness and low vision, for aids and advice, e.g. links to several national foundations are found at [www.visionaustralia.org.au/info.aspx?page=521 - ibo](http://www.visionaustralia.org.au/info.aspx?page=521#ibo) - Ensure appropriate spectacles. - To prevent falls, wearers of multifocal spectacles who regularly participate in outdoor activities should be provided with single lens glasses and counselling . |
| b230-b249 | Hearing functions | - Referral for specialist audiological or medical assessment. - Facilitate liaison with local/national foundation for hearing impaired, for aids and environmental adaptations such as specialised telephones. - Facilitate self-management of hearing aids. |
| b280 | Sensation of pain | - The British Geriatrics Society ‘The Assessment of Pain in Older People’,   [http://www.bgs.org.uk/Publications/Publication%20Downloads/Sep2007PainAssessment.pdf](http://www.bgs.org.uk/Publications/Publication Downloads/Sep2007PainAssessment.pdf) |
| b455 | Exercise tolerance functions*^b^* | - Address underlying health conditions. - Implement the Recommendations on physical activity for health for older Australians <http://www.health.gov.au/internet/main/publishing.nsf/Content/phd-physical-activity-adults-pdf-cnt.htm> - Other useful resources are:   The American College of Sports Medicine (ACSM) Position Stand, Exercise and Physical Activity for Older Adults <http://journals.lww.com/acsm-msse/Fulltext/2009/07000/Exercise_and_Physical_Activity_for_Older_Adults.20.aspx>  The World Health Organisation’s Global Recommendations on Physical Activity for Health.  <http://whqlibdoc.who.int/publications/2010/9789241599979_eng.pdf> |
| b6202 | Urinary continence | - Referral for specialist assessment. - NICE Guidelines ‘Urinary incontinence: the management of urinary incontinence in women’, <http://guidance.nice.org.uk/CG40> - SIGN Guideline for management of urinary incontinence in primary care, <http://www.sign.ac.uk/guidelines/fulltext/79/index.html> |
| b730 | Muscle power functions | - Muscle strength can be increased by resistance training . - Power training increases muscle power more effectively than progressive resistance training . - Principals of resistance training in older people are found within the ACSM position stand at <http://journals.lww.com/acsm-msse/Fulltext/2009/03000/Progression_Models_in_Resistance_Training_for.26.aspx>   Training may need to be modified in the frail person, to maintain safety.   - In frail older people, body weight may offer sufficient resistance. As strength increases, and if tolerated, additional resistance can be added using hand weights or weighted vests/belts. |
| b765 | Involuntary movement functions  *(e.g. impaired balance)* | - See d415 (Maintaining a body position) |
| Activities and participation | d410 | Changing basic body positions | - Assessment for and provision of equipment. |
| d415 | Maintaining a body position *(eg. unable to stand for long enough to complete a task, falling)* | - Appropriately designed exercise interventions are effective in reducing   rate and risk of falling in older people living in both the community and nursing care settings . Exercise should be ongoing, challenge balance and be undertaken at least two hours per week .   - Home safety interventions are effective in this high-risk frail group, and multifactorial falls assessment and intervention also reduce falls rate . - Replacing multifocal with single lens glasses reduces falls in people who regularly mobilise outdoors . - Overall hip protectors are not effective at reducing fractures and adherence is generally poor . However, wearing hip protectors at the time of a fall reduces the risk of hip fracture by 80% . Strategies to increase compliance include: education on outcomes associated with hip fracture, ensure correct size and reasonable comfort, match the brand hip protector with the person’s needs, provide extra pairs if incontinent, and minimise cost. |
| d430 | Lifting and carrying objects | - Target underlying problems, e.g. strengthen weak upper limb muscles, improve balance, intervention to reduce tremor. - Task specific practise, including structuring the environment to make carrying safe. - Education in relation to safe lifting techniques/back care - Trial equipment to facilitate moving objects. |
| d450  d455 | Walking  Moving around | - Advice about appropriate footwear. Shoes should have low heels and slip resistant outersoles. Narrow midsoles and very soft midsoles should be avoided. Shoes should be fitted firmly to the feet . Use manageable fasteners to ensure shoes are always done up firmly. - Provision of equipment and training in safe use. |
| d465 | Moving around using equipment | - We recommend using a walking aid when it is necessary to maintain safety and/or independence. Intervention should concurrently address the underlying cause of decreased balance/mobility. - Equipment is prescribed based upon assessment and tailored to the individual. Patients are trained in safe use of equipment and regular reassessment ensures equipment is progressed as ability improves or deteriorates. |
| d470 | Using transportation | - Access parking schemes for people with disability and taxi subsidy schemes. - Provide information regarding accessible public transport, assess and address barriers to using transport. - Access providers of community transport. |
| d530 | Toileting | - Common aids are toilet surrounds, raised toilet seats, grab rails by the toilet. - Task specific training as indicated. |
| d540 | Dressing | - Common strategies are: sit on a chair to dress, choose clothing that is easy to don and doff, and use aids to help put on socks and shoes. - Task specific training, with reference to the frail aged:   - Encourage or assist with laying out clothes in the order the person  should put them on (e.g. underwear first).  - Have loose-fitting, comfortable clothing; avoid girdles, control top  pantyhose, knee high nylons or tight bras. Short cotton socks and  loose cotton underwear are better choices.  - Sweat pants and shorts with elastic waistbands can be useful and  easily managed by the frail aged  - Velcro or large zipper pulls for clothing instead of shoelaces,  buttons or buckles will encourage independence  - Slip on shoes that won’t slip off or shoes with velcro straps are a  good alternative. Encourage wearing of clothes that make people feel good about themselves, as well as being practical and safe.   - Provide assistance as indicated. |
| d630 | Preparing meals  *(e.g. Inability to prepare meals / purchase food)* | - Provide information on purchase of pre-packaged meals from supermarket or home-delivery organisation. - Access delivery of groceries via home delivery services or internet shopping. - Facilitate access to and trial of home-delivered meals. |
| d640 | Doing housework | - Provision of services to assist with housework. |
| d620 | Acquisition of goods and services | - Access voluntary or funded services to assist with transport and/or shopping. - Education regarding internet shopping. |
| d760 | Family relationships | - Support carers effectively. Suggestions for assistance are given in Section 1.11 of The NICE clinical guideline “Dementia: Supporting people with dementia and their carers in health and social care” <http://guidance.nice.org.uk/CG42>. |
| d850-8555 | Remunerative and non-remunerative employment | - Encourage continuing engagement in meaningful paid and unpaid employment. |
| d910 | Community Life | - Assess goals regarding participation in community life and the associated barriers. - Facilitate access to community groups, day hospitals, religious meetings, and contact with family and friends, via referral, organisation of transport and physical support. |
| d920 | Recreation and leisure |
| Environmental contextual factors | e110 | Products or substances for personal consumption  *(e.g. medication)* | - Medication review, with the aim of minimising psychoactive medications. - Intervention can increase compliance with medications, e.g. tablets in blister packs, supportive care, reminders and self-monitoring . |
| e115, e120 | Products and technology for personal use in daily living, indoor and outdoor  mobility and transportation | - If practicable, home computers for email and word documents can enable older people to keep in touch with family. |
| e150, e155 | Design, construction and building products and technology  of buildings for pubic / private public use | - The principles of universal design and guidelines for home design can be accessed via <http://www.landcom.nsw.gov.au/downloads/uploaded/FINAL_Universal Housing Design Guidelines Fact Sheet_6507_740d.pdf> |
| e310, e320 | Immediate family, friends | - There should be early and ongoing engagement with support and education of formal and informal carers . Caregivers and family should be taught about frailty, interventions to optimise function and be involved in planning and development of management plans. - Assess needs of carers - Facilitate access to Carers Associations - Facilitate assessment for respite - Information about needs and support of carers in Australia is found at <http://www.aph.gov.au/house/committee/fchy/carers/index.htm> |
| e325 | Acquaintances, peers, colleagues, neighbors and community members  *(e.g. social isolation)* | - Encourage older people to maintain contact with as many people as possible, from a variety of social networks. |
| e340 | Personal care providers and personal assistants | - Community services should be provided in a coordinated manner, consistent with available community care packages. |
| e355/360 | Health professionals and health-related professionals | - Every frail individual should receive primary health care from a defined health professional. - A comprehensive health assessment may identify health conditions that are preventable or treatable, for example The Australian Over 75 Health Assessment <http://www.health.gov.au/internet/main/publishing.nsf/Content/mbsprimarycare_mbsitem_75andolder> - A range of health professionals should be involved, e.g. podiatrist, physiotherapist, occupational therapist, community nurse. |
| e410/e420 | Individual attitudes of immediate family members, friends | - Educate family and friends about the dynamic nature of frailty, the potential to mitigate the condition and interventions in which they can be involved. |
| e430/e440/  e450/e455 | Individual attitudes of people in positions of authority/personal care providers and  personal assistants/ health professionals /health related professionals / | - Health professionals should liaise closely with aged care facilities, regarding the nature of frailty, ongoing reassessment, intervention strategies and respite care. - Education for health professionals regarding the condition of frailty, the patterns and interventions. |
| e460 | Societal attitudes | - Support the United Nations Convention of the Rights of Persons with Disabilities http://www.un.org/disabilities/ |
| e540 | Transportation services, systems and policies | - See d470 (Using transportation) |
| e575 | General social support services, systems and policies | - Present options for, and facilitate access to, organisations providing personal alarms, meal delivery, daily telephone contact, gardening services etc. |
| e580 | Health services, systems and policies | - Facilitate entry to respite/day centres as appropriate. - Provision and co-ordination of services, with preference given to packages of care, followed by single services, followed by a residential aged care facility. The case co-ordinator must ensure the frail individual and their family/carers understand the services provided and how to promptly access greater assistance in times of increased need. |
| Personal contextual factors |  | Handling stress |  |
|  | Coping skills |  |
|  | Fear |  |
|  | Self efficacy | - Strategies to facilitate behavior change to enhance participation in intervention programs are outlined in the NICE Guidance <http://www.nice.org.uk/PH6> |
|  | Grief |  |
|  | Lack of resilience |  |
| Concepts not identified in the ICF. |  | Self rated health |  |
|  | Satisfaction with health |  |
|  | Effect of major trauma throughout lifespan |  |
|  | Expectation about how health will change in the future | - Education about prognosis - Discuss Enduring Power of Attorney |
|  | Perception of how health limits functional tasks |  |

*^a^* The ICF does not provide specific codes or domains for the ICF levels of health conditions and personal contextual factors

*^b^* Frailty Phenotype criteria.
